# A Randomized Trial of Selenium Supplementation and Risk of Type-2 Diabetes, as Assessed by Plasma Adiponectin

**DOI:** 10.1371/journal.pone.0045269

**Published:** 2012-09-19

**Authors:** Margaret P. Rayman, Gabrielle Blundell-Pound, Roberto Pastor-Barriuso, Eliseo Guallar, Holger Steinbrenner, Saverio Stranges

**Affiliations:** 1 Faculty of Health and Medical Sciences, University of Surrey, Guildford, United Kingdom; 2 National Center for Epidemiology, Carlos III Institute of Health and Consortium for Biomedical Research in Epidemiology and Public Health (CIBERESP), Madrid, Spain; 3 Johns Hopkins University Bloomberg School of Public Health, Baltimore, Maryland, United States of America; 4 National Center for Cardiovascular Research (CNIC), Madrid, Spain; 5 Institute for Biochemistry and Molecular Biology I, Heinrich-Heine-University Duesseldorf, Duesseldorf, Germany; 6 Division of Health Sciences, University of Warwick Medical School, Coventry, United Kingdom; Johns Hopkins University Bloomberg School of Public Health, United States of America

## Abstract

**Background:**

Evidence that selenium affects the risk of type-2 diabetes is conflicting, with observational studies and a few randomized trials showing both lower and higher risk linked to the level of selenium intake and status. We investigated the effect of selenium supplementation on the risk of type-2 diabetes in a population of relatively low selenium status as part of the UK PRECISE (PREvention of Cancer by Intervention with SElenium) pilot study. Plasma adiponectin concentration, a recognised independent predictor of type-2 diabetes risk and known to be correlated with circulating selenoprotein P, was the biomarker chosen.

**Methods:**

In a randomized, double-blind, placebo-controlled trial, five hundred and one elderly volunteers were randomly assigned to a six-month intervention with 100, 200 or 300 µg selenium/d as high-selenium or placebo yeast. Adiponectin concentration was measured by ELISA at baseline and after six months of treatment in 473 participants with one or both plasma samples available.

**Results:**

Mean (SD) plasma selenium concentration was 88.5 ng/g (19.1) at baseline and increased significantly in the selenium-treatment groups. In baseline cross-sectional analyses, the fully adjusted geometric mean of plasma adiponectin was 14% lower (95% CI, 0–27%) in the highest than in the lowest quartile of plasma selenium (*P* for linear trend = 0.04). In analyses across randomized groups, however, selenium supplementation had no effect on adiponectin levels after six months of treatment (*P* = 0.96).

**Conclusions:**

These findings are reassuring as they did not show a diabetogenic effect of a six-month supplementation with selenium in this sample of elderly individuals of relatively low selenium status.

**Trial Registration:**

Controlled-Trials.com ISRCTN25193534

## Introduction

The relationship between selenium (Se) and type-2 diabetes is a conundrum. That a relationship should exist is unsurprising given the clear link found between diabetes or insulin resistance and a number of selenoproteins in both human and animal studies [1]–[7]. Results from epidemiological studies on Se and type-2 diabetes are conflicting. Higher serum Se concentration was associated with a higher prevalence of diabetes in several cross-sectional studies [8]–[11]. However, longitudinal studies have not supported a causal role for Se in type-2 diabetes [10], [12]; indeed, high plasma Se was associated with a decreased risk of onset of hyperglycemia over a nine-year follow-up period among male participants in the prospective French EVA study [12].

Results of randomised trials have also been inconclusive. The Nutritional Prevention of Cancer (NPC) trial, carried out in the eastern US, showed a significantly increased risk of type-2 diabetes in those supplemented with Se (200 µg/day as Se-yeast) over an average period of 7.7 years [13]. The increased risk was driven by those in the highest tertile of plasma Se at baseline. By contrast, in the large Selenium and Vitamin E Cancer Prevention Trial (SELECT), there was a small non-significant increase in the number of cases of adult-onset diabetes in subjects supplemented with Se alone (200 µg/day as selenomethionine) [14] that diminished further on follow-up for an additional 18 months [15].

To advance our understanding of the effect of Se on the risk of type-2 diabetes, we used stored plasma samples from the UK PRECISE (PREvention of Cancer by Intervention with SElenium) pilot study to test the effect of Se supplementation on plasma adiponectin, a strong independent predictor of type-2 diabetes risk [16]–[20]. Adiponectin sensitizes skeletal muscle and liver to the action of insulin and stimulates glucose uptake *via* the cellular fuel sensor, AMP-activated protein kinase (AMPK) [21]; [22]. Adiponectin has been linked to Se or selenoproteins in a number of ways, though as with the epidemiology, the relationship is not straightforward: (i) circulating selenoprotein P was negatively associated with circulating adiponectin in patients with type-2 diabetes [5]; (ii) patients with markedly reduced expression of selenoproteins due to a rare defect in the *SECISBP2* gene had elevated blood adiponectin and enhanced insulin signalling [23]; (iii) selenoprotein P knock-out mice had significantly higher blood adiponectin levels than wild-type mice [5]; (iv) Se supplementation of macrophages increases the production of 15-deoxy-Delta12,14-prostaglanin J2 (15d-PGJ2), an activator of peroxisome proliferator-activated nuclear receptor-γ (PPAR-γ) [24]; this is relevant because PPAR-γ agonists have been shown to increase the expression and protein levels of adiponectin [25], [26]; (v) knock-down of selenoprotein P in adipocytes markedly lowered the expression of both adiponectin and PPAR-γ [27]. Furthermore, both Se/selenoprotein P and adiponectin are associated with raised HDL cholesterol [20], [28], [29] and reduced inflammation [20], [28], [30], and both can affect AMPK, though in opposite directions [4], [22]. Most importantly for our study, adiponectin is a useful biomarker of type-2 diabetes risk in non-fasted plasma samples, which ours are, as diurnal variability is minor and there is no noticeable effect of food intake [16], [31], [32].

In PRECISE, 501 elderly volunteers were randomly assigned to a six-month intervention with 100, 200 or 300 µg Se/d as high-Se or placebo yeast [29], [33], [34]. PRECISE participants come from a population of relatively low Se status where antioxidant selenoproteins such as glutathione peroxidase and selenoprotein P are unlikely to be optimised at baseline. We hypothesised that the 100 µg dose might increase plasma adiponectin concentration (indicative of a reduced risk of type-2 diabetes) by optimising selenoprotein activity, whereas the much higher 300 µg dose might potentially have an adverse effect.

## Methods

The protocol for this trial and supporting CONSORT checklist are available as supporting information; see Checklist S1 and Protocol S1.

### Ethics Statement

The study had approval from UK Local Research Ethics Committees [South Tees (ref: 99/69), Worcestershire Health Authority (ref: LREC 74/99), Norwich District (ref: LREC 99/141), Great Yarmouth and Waveney (under reciprocal arrangements with Norwich District LREC)] and participants provided written informed consent.

### Design and Study Population

The UK pilot study for the planned international PRECISE (Prevention of Cancer by Intervention with Selenium) trial was designed to assess the viability of conducting the trial in the UK. The UK PRECISE pilot (ISRCTN 25193534) was a double-blind, placebo-controlled, four-arm parallel-group study, stratified by age and sex [29], [33], [34]. The target accrual (501 subjects in 12 months) was chosen to give sufficient subjects to draw reasonable inferences about recruitment, compliance and loss to follow-up.

Volunteers were recruited from four general practices (study centers) in different parts of the country (see **Table 1**) affiliated with the Medical Research Council (MRC) General Practice Research Framework. Between June 2000 and July 2001, research nurses recruited similar numbers of men and women from each of three age groups: 60–64, 65–69 and 70–74 years. Exclusion criteria were: i) a Southwest Oncology Group performance status score >1 (i.e. incapable of carrying out light housework or office work); ii) active liver or kidney disease; iii) prior diagnosis of cancer (excluding non-melanoma skin cancer); iv) diagnosed HIV infection; v) on immunosuppressive therapy; vi) diminished mental capacity; vii) taking ≥50 µg/day of Se supplements in the previous six months (by patient report).

**Table 1:** Descriptive baseline characteristics overall and by treatment group*

				Selenium dose (µg/d)			
Characteristic	Availabledata	Overall	Placebo	100	200	300	*P*value†
Participants		473 (100.0)	112 (23.7)	120 (25.4)	124 (26.2)	117 (24.7)	
Study center	473						0.99
Bungay (eastern England)		117 (24.7)	29 (25.9)	29 (24.2)	31 (25.0)	28 (23.9)	
Guisborough (northeast England)		187 (39.5)	44 (39.3)	49 (40.8)	48 (38.7)	46 (39.3)	
Bromsgrove (central England)		112 (23.7)	25 (22.3)	28 (23.3)	31 (25.0)	28 (23.9)	
Linthorpe (northeast England)		57 (12.1)	14 (12.5)	14 (11.7)	14 (11.3)	15 (12.8)	
Age (years)	471	67.5 (4.1)	67.6 (4.2)	67.3 (4.1)	67.3 (4.0)	67.7 (4.1)	0.82
Sex	473						0.88
Men		249 (52.6)	58 (51.8)	60 (50.0)	68 (54.8)	63 (53.8)	
Women		224 (47.4)	54 (48.2)	60 (50.0)	56 (45.2)	54 (46.2)	
Smoking status	473						0.70
Never		206 (43.6)	53 (47.3)	55 (45.8)	54 (43.5)	44 (37.6)	
Former		222 (46.9)	51 (45.5)	53 (44.2)	56 (45.2)	62 (53.0)	
Current		45 (9.5)	8 (7.1)	12 (10.0)	14 (11.3)	11 (9.4)	
Drinking habits	473						0.54
Never		28 (5.9)	6 (5.4)	8 (6.7)	6 (4.8)	8 (6.8)	
Former		34 (7.2)	6 (5.4)	6 (5.0)	14 (11.3)	8 (6.8)	
Current		411 (86.9)	100 (89.3)	106 (88.3)	104 (83.9)	101 (86.3)	
Body mass index (kg/m^2^)	471	27.5 (5.0)	27.4 (4.2)	27.8 (4.3)	27.4 (4.5)	27.6 (6.6)	0.91
Waist circumference (cm)	471	96.2 (13.8)	95.8 (13.3)	96.4 (14.7)	96.2 (12.4)	96.3 (14.9)	0.99
Total cholesterol level (mmol/L)	439	5.98 (1.07)	6.00 (1.03)	6.11 (1.18)	5.98 (1.03)	5.84 (1.01)	0.30
HDL cholesterol level (mmol/L)	439	1.61 (0.37)	1.65 (0.42)	1.59 (0.33)	1.59 (0.32)	1.63 (0.41)	0.59
Use of lipid-lowering medication	473	24 (5.1)	9 (8.0)	4 (3.3)	3 (2.4)	8 (6.8)	0.15
Use of diabetes medication	473	17 (3.6)	5 (4.5)	3 (2.5)	4 (3.2)	5 (4.3)	0.83
Plasma selenium level (ng/g)	451	88.5 (19.1)	88.3 (19.0)	87.3 (17.9)	88.1 (19.7)	90.2 (19.6)	0.72
Plasma adiponectin level (µg/mL)	431						
Arithmetic mean (SD)		9.50 (5.07)	9.64 (5.05)	8.85 (4.54)	8.98 (4.86)	10.56 (5.66)	0.05
Geometric mean		8.07	8.26	7.51	7.69	8.92	0.16
Median (25th to 75th percentiles)		8.55	8.59	8.36	7.34	9.76	
		(5.74 to 12.70)	(5.99 to 13.15)	(5.66 to 10.54)	(5.21 to 12.70)	(6.12 to 14.78)	

* Data are means (SDs) or numbers (%) in participants with at least one adiponectin measurement available either at baseline or at six months.

† *P* values for homogeneity of means or proportions across the four treatment groups, as obtained from one-way analysis-of-variance *F* tests for continuous variables and Pearson’s chi-squared tests for categorical variables.

HDL, high-density lipoprotein.

### Randomization and Interventions

Computer-generated random permuted blocks, stratified by study center, gender and age group were used to generate the randomization list at the Clinical Trials and Statistics Unit, Institute of Cancer Research, Sutton Surrey, UK. Following a four-week placebo run-in, 501 volunteers were randomly assigned (allocation ratio 1∶1:1∶1) to one of four treatment regimens: placebo, 100, 200 or 300 µg of Se per day for a minimum of six months. The intervention agent was high-Se yeast, SelenoPrecise™ (Pharma Nord, Vejle, Denmark), or an identical placebo yeast (comprising 250 mg yeast placebo, 80 mg cellulose, 65 mg dicalcium phosphate, and ≤5 mg of other inactive ingredients). Participants, research nurses, other study center personnel, investigators and those who analyzed the data were blinded to treatment.

### Data Collection and Follow-up

**Figure 1:**
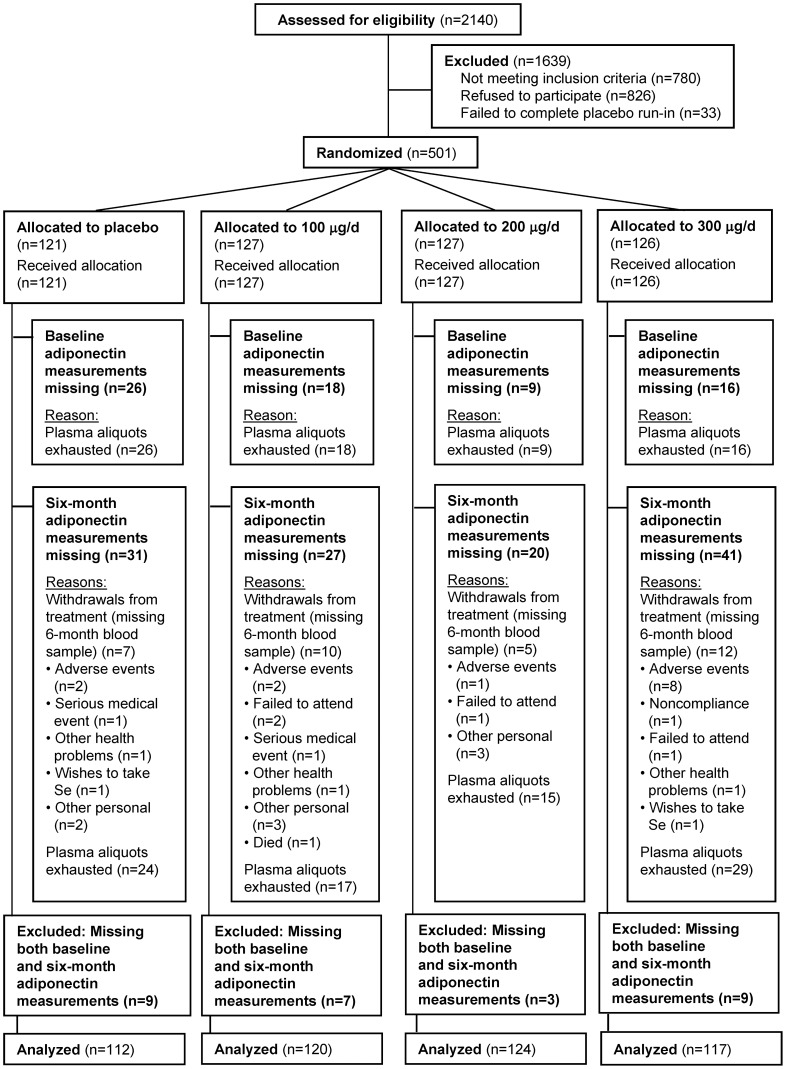
CONSORT flow diagram.

Demographic data, medical history, and other health-related information, including medication and supplement use, were collected at baseline. Of 501 randomised participants, 34 withdrew from treatment (**Figure 1**). However, there was no statistically significant difference in numbers of participants withdrawing across treatment groups (*P* from Pearson’s chi-squared test  = 0.31).

Participants provided a non-fasting blood sample at both baseline and six months. Heparinised plasma was prepared and frozen at the study centers. Plasma samples were transferred to the University of Surrey on dry ice where they were stored at −80°C. At the six-month follow-up appointment, questionnaires were used to see if there had been any new symptoms or illnesses since randomization and whether use of medication and supplements had changed. Compliance with randomised treatment was determined by pill count. Participants were considered compliant if they took at least 80% of their allocated tablets. In addition, each participant’s plasma Se was compared with the mean of the group to detect non-compliant participants or “drop-ins”. Reasons for participant withdrawal were noted.

The last planned six-month follow-up visit (with blood draw) was in January 2002. However, volunteers, who were to have been the first cohort of the main PRECISE trial, continued treatment and six-month follow-up visits until mid-2003 when it became clear that the international study was not going to be funded.

### Selenium Measurement

Lithium-heparin plasma was analysed for Se at Central Science Laboratory, Sand Hutton, UK, by hydride-generation Inductively-Coupled-Plasma Mass Spectrometry as previously described. Quality control procedures accredited under the UK Accreditation Scheme were followed. Accuracy was assured by good performance on the analysis of certified reference materials [34].

### Adiponectin Measurement

Total plasma adiponectin concentration was measured at baseline and at the six-month follow-up in 473 participants who had one or both plasma samples available using 4.5 hour solid-phase ELISA kits (Quantikine®, Human Total Adiponectin/Acrp30 Immunoassay, R&D systems, Abingdon, OX14 3NB, UK); the detailed protocol accompanying the kits was followed (http://www.rndsystems.com/pdf/drp300.pdf ). The intra- and inter-assay coefficients of variation were 3.4% and 8.2%. Characteristics of participants with and without adiponectin measurements available did not differ (data not shown).

### Statistical Methods

For the analyses of the randomized groups, all trial participants for whom plasma adiponectin measurements were available at the baseline or follow-up visits were assigned to their randomized treatment group, irrespective of compliance. Plasma adiponectin levels were right-skewed and log-transformed for the analyses. The effect of Se supplementation was estimated by using linear mixed models on log-transformed adiponectin levels with fixed treatment-by-time interactions and random between-subject variations in both baseline adiponectin levels (intercepts) and adiponectin changes over time (slopes) in 473 participants with at least one adiponectin measurement available either at baseline or at six months. Models were adjusted for sex and study center. Reverting model results to the original scale, we estimated the ratio of geometric mean adiponectin concentrations at six months to those at baseline for each treatment group, as well as the sex- and center-adjusted relative ratios of adiponectin change for the three active treatment groups compared to placebo (treatment effect). For the average sample size available per treatment group and an uncorrected two-sided alpha level of 0.05, the power of the study to detect an underlying 20% change in geometric mean adiponectin concentrations comparing any Se supplementation group to placebo was 77.1%.

In sensitivity analyses restricted to 340 participants with adiponectin measurements available both at baseline and at six months, standard analysis of covariance models relating log-transformed adiponectin concentrations at six months to treatment assignment adjusting for log-transformed adiponectin levels at baseline, sex, and study center yielded similar results (data not shown). To evaluate differential treatment effects by sex, all two- and three-way interactions among time, treatment group, and sex were included as fixed effects in the above mixed model. P-values for treatment-by-sex interactions were obtained from Wald tests for the joint null hypothesis that all three-way interaction coefficients were simultaneously zero.

In addition to estimating Se supplementation efficacy from the trial intervention results, we evaluated the cross-sectional association between plasma Se concentrations and adiponectin levels at baseline. Using linear regression models on log-transformed adiponectin levels, we estimated the multivariable-adjusted geometric mean ratios in baseline adiponectin levels for a 50-ng/g increase in baseline Se levels, as well as for the three highest quartiles of baseline Se compared with the lowest quartile. Tests for linear trend across quartiles were conducted by including in linear regression models an ordinal variable with the median baseline Se level of each quartile. We used three models with progressive degrees of adjustment. Model 1 adjusted for age, sex, and study center; model 2 further adjusted for smoking, drinking, body mass index, and waist circumference; and model 3 further adjusted for total cholesterol, HDL cholesterol, lipid-lowering and diabetes medications. Differences in baseline association by sex were evaluated using interaction terms between baseline Se and sex.

The reported *P* values were two-sided and not adjusted for multiple testing. Statistical analyses were performed with Stata, version 12 (StataCorp, College Station, Texas).

## Results

### Baseline Characteristics

Overall mean (SD) plasma Se at baseline was 88.5 (19.1) ng/g [equivalent to 90.8 (19.6) µg/L] [35]. Baseline adiponectin levels displayed high variability within the population [mean (SD) 9.50 (5.07) µg/mL]. There were no statistically significant differences between treatment groups at baseline in plasma Se concentrations (*P* = 0.72) or in other participant characteristics (**Table 1**).

### Cross-sectional Association between Plasma Selenium and Adiponectin Concentrations

At baseline, the geometric means of plasma adiponectin for quartiles 1 through 4 of plasma Se were 8.30, 8.14, 7.71, and 7.79 µg/mL, respectively (**Table 2**). In models adjusted for age, sex, and study center, the geometric mean of plasma adiponectin was 7% lower (95% CI, 21% lower to 9% higher) in the highest compared to the lowest quartile of plasma Se (*P* for linear trend  = 0.36). In fully adjusted models, the geometric mean of plasma adiponectin was 14% lower (95% CI, 0 to 27%) in the highest than in the lowest quartile of plasma Se (*P* for linear trend  = 0.04). Lipid levels (and particularly HDL cholesterol) were the main covariates responsible for increasing the strength of the association between the basic and fully adjusted models. The inverse association between Se and adiponectin observed at baseline was evident in men and women (*P* for interaction between Se and sex  = 0.93; **Table S1**).

**Table 2:** Cross-sectional association between plasma selenium and adiponectin concentrations at baseline*

		Baseline plasma selenium quartile (ng/g)				
	50-ng/g increase in baseline selenium level	First (48.6 to 75.0)	Second (75.1 to 88.0)	Third (88.1 to 100.0)	Fourth (100.1to 177.0)	*P* value for trend∥
Participants (n)	419	106	106	105	102	
Median baseline selenium level (ng/g)	88.0	66.9	81.2	92.9	109.0	
**Baseline adiponectin level (** **µg/mL)**						
Geometric mean (SD)	7.99 (1.86)	8.30 (1.73)	8.14 (1.81)	7.71 (1.76)	7.79 (2.13)	
Geometric mean ratio (95% CI)						
Model 1†	1.00	1	1.03	1.02	0.93	0.36
	(0.86 to 1.17)	(Reference)	(0.88 to 1.21)	(0.88 to 1.20)	(0.79 to 1.09)	
Model 2‡	0.99	1	1.01	1.01	0.91	0.28
	(0.85 to 1.15)	(Reference)	(0.86 to 1.18)	(0.87 to 1.19)	(0.78 to 1.07)	
Model 3§	0.90	1	1.00	0.95	0.86	0.04
	(0.78 to 1.04)	(Reference)	(0.86 to 1.16)	(0.82 to 1.10)	(0.73 to 1.00)	

* Results were obtained from linear regression models of log-transformed adiponectin levels on selenium levels using only cross-sectional data from the

baseline visit.

† Model 1 adjusted for age (continuous), sex, and study center (Bungay, Guisborough, Bromsgrove, or Linthorpe).

‡ Model 2 further adjusted for smoking status (never, former, or current), drinking habits (never, former, or current), body mass index (continuous), and waist circumference (continuous).

§ Model 3 further adjusted for total cholesterol level (continuous), HDL cholesterol level (continuous), use of lipid lowering medications, and use of diabetes medications.

∥*P* values for linear trend were obtained from Wald tests for the coefficient of an ordinal variable with the median baseline selenium level of each quartile in linear regression models.

### Analysis of Randomized Groups

Ninety-four percent of the 473 participants missed less than 10% of the total number of study tablets according to pill count. After six months of supplementation, plasma Se had increased significantly and proportionally to the assigned dose in the three active treatment groups but was unchanged in the placebo group. Adiponectin levels, however, remained virtually unchanged after six months of intervention in the four treatment groups (**Table 3**). Adjusting for longitudinal changes in the placebo group, geometric mean adiponectin levels decreased by 4% (95% CI, 18% lower to 13% higher) after six months of Se supplementation at 100 µg/d, decreased by 1% (95% CI, 15% lower to 16% higher) after supplementation at 200 µg/d, and remained unchanged (95% CI, 15% lower to 18% higher) after supplementation at 300 µg/d (overall *P* for the three active treatment groups compared to placebo  = 0.96).

**Table 3:** Effect of selenium supplementation on changes in plasma adiponectin and selenium concentrations after six months*

		Selenium dose (µg/d)			
Variable	Placebo	100	200	300	*P* value‡
**Plasma adiponectin level (** **µg/mL)**					
Geometric mean (SD) at baseline	8.26 (1.81)	7.51 (1.96)	7.69 (1.79)	8.92 (1.87)	
Geometric mean (SD) at 6 mo	8.16 (1.96)	7.49 (2.19)	7.67 (1.82)	8.99 (1.78)	
Ratio at 6 mo to baseline	1.01	0.97	1.00	1.01	
(95% CI)	(0.89 to 1.13)	(0.87 to 1.08)	(0.90 to 1.11)	(0.89 to 1.13)	
Relative ratio	1	0.96	0.99	1.00	0.96
(95% CI)	(Reference)	(0.82 to 1.13)	(0.85 to 1.16)	(0.85 to 1.18)	
*P* value†		0.66	0.92	0.99	
**Plasma selenium level (ng/g)**					
Arithmetic mean (SD) at baseline	88.3 (19.0)	87.3 (17.9)	88.1 (19.7)	90.2 (19.6)	
Arithmetic mean (SD) at 6 mo	90.2 (26.8)	143.9 (25.9)	188.1 (42.9)	225.9 (52.4)	
Change from baseline to 6 mo	2.1	57.8	100.3	136.4	
(95% CI)	(−4.9 to 9.0)	(51.0 to 64.6)	(93.6 to 106.9)	(129.2 to 143.6)	
Difference in change	0	55.8	98.2	134.3	<0.001
(95% CI)	(Reference)	(46.1 to 65.5)	(88.6 to 107.8)	(124.4 to 144.3)	
*P* value†		<0.001	<0.001	<0.001	

* Results were obtained from linear mixed models on log-transformed adiponectin levels (and untransformed selenium levels) with fixed treatment-by-time interactions and random between-subject variations in both baseline levels (intercepts) and longitudinal changes over time (slopes).

† *P* values comparing the ratio of geometric mean adiponectin levels (and the change in arithmetic mean selenium levels) at six months to baseline in each active treatment group to placebo, as obtained from Wald tests for each treatment-by-time interaction coefficient in linear mixed models.

‡ Overall *P* value comparing the three active treatment groups to placebo, as obtained from the joint Wald test for all treatment-by-time interaction coefficients in linear mixed models.

The null effect of Se supplementation on adiponectin levels did not differ significantly by sex (*P* for treatment-by-sex interaction  = 0.39; **Table S2**). In additional subgroup analyses, trial results remained virtually unchanged after excluding 17 participants that used diabetes medications at baseline (data not shown) and there were no statistically significant differences across study centers, or by category of body mass index, baseline plasma Se concentrations, or baseline adiponectin concentrations (data not shown).

### Adverse Events

No serious adverse events occurred. Twelve adverse events were reported, which were principally stomach or abdominal discomfort. These were equally associated with Se or placebo and were not dependent on dose (data not shown).

## Discussion

To our knowledge this is the first randomized trial to examine the effect of supplementation with Se as a single nutrient on a biomarker of type-2 diabetes risk in a population of relatively low Se status. In this study, we observed an inverse cross-sectional association between plasma Se and adiponectin concentrations at baseline, but Se supplementation for six months over a wide range of doses had no effect on plasma adiponectin concentration. As insulin resistance can be triggered by oxidative stress and ameliorated by antioxidant treatment [36], we might have expected some benefit of Se supplementation in our population where Se status was rather low. In a population of similar Se status to ours, higher baseline Se status did appear to protect against the onset of hyperglycemia over a nine-year follow-up period, though only among male participants [12]. In our study population, few participants would have had maximized activities or concentrations of selenoproteins; animal studies have shown that not only excessive levels of GPx1 but also low levels of GPx1 and other “stress-related” selenoproteins can cause insulin resistance and hyperglycaemia [37]. However, we found null effects of Se supplementation not only overall, but also in subgroups defined by gender, body mass index category, and baseline Se concentrations. In a small trial in pigs, which are a good model for human metabolism, no apparent increase in molecular markers of insulin resistance was observed in adipose tissue after 16 weeks of dietary supplementation with supranutritional Se [38].

It could be argued that our population was insufficiently obese (BMI 27.5 kg/m^2^) for adiponectin levels to rise in response to Se supplementation [39]. However, a number of studies have found significantly lowered adiponectin levels in those with low or normal overall fatness who have characteristics of the metabolic syndrome such as insulin resistance [40], [41].

Cross-sectional studies have previously found a positive association between serum/plasma Se and type-2 diabetes or fasting plasma glucose [8]–[11]. Furthermore, serum selenoprotein P, a major component of Se in plasma, has been shown to be negatively associated with serum adiponectin [5]. Our finding of a negative association between plasma Se and adiponectin at baseline accords with those results. The cross-sectional associations between plasma Se, selenoprotein P and diabetes risk could be explained by the linked expression of selenoprotein P and gluconeogenic enzymes that promote the *de novo* biosynthesis of glucose [3], [7]. Thus significant correlations have been found between serum selenoprotein P and adiponectin [5], fasting plasma glucose [4] and HbA1c [4] while circulating selenoprotein P concentration was significantly higher in people with type-2 diabetes or pre-diabetes than in those with normal glucose tolerance [4]–[6]. This cross-sectional association, however, could be driven by plasma glucose rather than by high Se; as an example, the cultivation of hepatocytes in hyperglycaemic medium significantly increased selenoprotein P secretion and mRNA levels [3], [4].

How do the results of our trial sit in the context of previous trial findings? Participants in the NPC trial had a significantly increased risk of type-2 diabetes on supplementation with 200 µg Se/d, the effect being driven by those in the top Se tertile at baseline [13]. This may have resulted from an adverse effect of Se on insulin signalling, acting through raised plasma selenoprotein P and decreased inhibition of the phosphatase, PTP-1B, known to antagonise insulin signalling [4], [42], [43]. At baseline, one third of the participants in the NPC trial had plasma Se >121.6 µg/L (highest tertile of the Se distribution) whereas that level was reached in only 5.8% of PRECISE participants. Our results are thus comparable to those of participants in the lower tertiles of the NPC trial in whom no significant effect was seen [13]; the adverse effects of additional Se in some participants of both PRECISE and the NPC trial may have been balanced by the achievement of an adequate level of GPx1 and other “stress-related” selenoproteins in others [37].

As in PRECISE, Se supplementation had no effect on the risk of type-2 diabetes in SELECT (RR 1.07, 99% CI 0.94–1.22 [14]; RR 1.04, 99% CI 0.91–1.18 [15]). This similarity is at first surprising as SELECT participants had a much higher baseline Se status than those in PRECISE and even than those in the top tertile of the NPC trial (mean/median serum/plasma Se, 136 *vs* 91 and 122 µg/L, respectively) [13], [14]. In SELECT, unlike PRECISE or indeed the top tertile of NPC, the expression or concentration of selenoprotein P may already have reached a plateau [44] or passed a threshold of risk prior to supplementation in almost all participants. Thus if an increase in selenoprotein P concentration is the cause of increased type-2 diabetes risk as suggested by some authors [4]–[7], no adverse effect of additional Se would have been seen, as was indeed the case. The existence of a U-shaped association between selenoprotein activity/concentration and type-2 diabetes risk might explain some of the apparently contradictory findings [45].

Another possible reason for a lack of effect of Se supplementation on adiponectin (PRECISE) or type-2 diabetes (SELECT) is that Se or selenoprotein P does not cause an increased risk of type-2 diabetes or a fall in circulating adiponectin. To date, apart from the findings in the top tertile of the NPC trial, which derive from a *post-hoc* analysis of a small trial, all the evidence linking Se or selenoprotein P to type-2 diabetes is cross-sectional. The conflicting observations that selenoprotein P knock-out mice had higher blood adiponectin levels than wild-type mice [5], and that knock-down of selenoprotein P in adipocytes markedly lowered the expression of adiponectin [27], do not fit with a causal relationship, nor can such a relationship explain the opposite effects of selenoprotein P and adiponectin on AMPK, a positive regulator of insulin synthesis [4], [22]. Although Misu and colleagues found a correlation between selenoprotein P and circulating adiponectin in 36 type-2 diabetics, it was not strong, explaining only 13% of the variance in adiponectin concentration [5]. Furthermore, they found no relationship between selenoprotein P and QUICKI (quantitative insulin sensitivity index), a marker of insulin resistance [5]. Evidence from two small interventions also fails to support a diabetogenic effect of Se; one study found no significant disturbances in plasma glucose after six weeks of supplementation with 150 µg/d Se as dairy- or yeast-Se [46] while the other, a randomized, controlled trial, found that 200 µg Se/d as yeast-Se for six weeks significantly lowered fasting serum insulin and HOMA-IR (homeostasis model assessment of insulin resistance) [47].

A major limitation of our study was the high variability of plasma adiponectin concentrations. In spite of this, we found a significant association between Se and adiponectin levels at baseline and the power of our analysis of randomized treatment groups to detect an underlying difference of 20% in geometric mean adiponectin concentrations between the Se supplementation groups and placebo was 77.1%. Follow-up was only for six months which may not have been long enough to see an effect. However, adiponectin can increase significantly within weeks in response to treatment with PPAR-γ ligands such as the thiazolidinediones, even in lean subjects [48]. As Se supplementation has been shown to increase the synthesis of a PPAR-gamma activator [24], Se-supplementation might have had a fairly rapid effect by that mechanism. Lastly, the age-range of the participants was restricted (60–74y), and our findings may not apply to younger age groups.

In summary, we found an inverse association of Se and adiponectin concentrations at baseline but no effect of supplementation for six months with 100, 200 or 300 µg/d of Se as Se-yeast on plasma adiponectin concentrations. Given the positive cross-sectional associations seen between biomarkers of Se and type-2 diabetes and the adverse effect seen in the NPC trial in a US population, our results are reassuring, at least for populations of equivalent Se status to ours i.e. those of other European countries. However, as we measured plasma adiponectin as a biomarker and not glucose or insulin and our trial only lasted for six months, additional research is needed to characterize fully the role of Se in diabetes risk.

## Supporting Information

Checklist S1 **CONSORT Checklist.**
(DOC)

Protocol S1 **Trial Protocol.**
(RTF)

Table S1 **Cross-sectional association between plasma selenium and adiponectin concentrations at baseline by sex.**
(DOC)

Table S2 **Effect of selenium supplementation on changes in plasma adiponectin concentrations after six months by sex.**
(DOC)
