# Supplementary material for: A Randomized Trial of Selenium Supplementation and Risk of Type-2 Diabetes, as Assessed by Plasma Adiponectin
Source: PLoS One. 2012 Sep 19;7(9):e45269. doi: 10.1371/journal.pone.0045269 (PMC3446875; doi:10.1371/journal.pone.0045269)
Supplement: Protocol S1 — Trial Protocol. (RTF) [file pone.0045269.s002.rtf]

THE PRECiSE TRIAL

PREvention of Cancer by Intervention with Selenium


A randomised, double-blind, placebo-controlled cancer prevention trial, with an estimated duration of 5 years and 
with 52,000 subjects recruited from the general populations 
of the United Kingdom, Denmark, Sweden, Finland and 
the United States


THE UK PILOT STUDY


PROTOCOL – January 2000


THE PRECiSE TRIAL

PREvention of Cancer by Intervention with Selenium

THE UK PILOT STUDY

STUDY PERSONNEL

Dr. Margaret Rayman	UK Cohort Leader, Selenium scientist 
	University of Surrey, Guildford	
Dr. Ross Lawrenson	Epidemiologist	University of Surrey, Guildford
	
Professor Hilary Thomas 	Oncologist 	University of Surrey and Royal Surrey County Hospital, Guildford
	
Ms. Judith Bliss	Statistician	Clinical Trials & Statistics Unit, Institute of Cancer Research
	
Miss Rachel Galassini	Study manager	University of Surrey, Guildford	

COLLABORATORS

Dr Madge Vickers	MRC General Practice Research Framework Manager	
Ms. Jeannett Martin	Chief Nurse, MRC General Practice Research Framework 	
Prof. Larry Clark 	Principal Investigator – PRECISE Trial	
Dr. Jim Marshall	Co-Principal Investigator – PRECISE Trial & US Cohort Director 	
Dr. Kim Overvad	European Cohort Director
	
Dr Soeren Cold	Danish Cohort Director	
Prof. Hans-Olov Adami 	Swedish Cohort Director	
Prof. Jarmo Virtamo	Finnish Cohort Director	
Mr. Sven Moesgaard
	Director, Pharma Nord	
		
For further information contact
Dr. Margaret Rayman																					Rachel Galassini
Centre for Nutrition and Food Safety													School of Biological Sciences
School of Biological Sciences																	University of Surrey
University of Surrey																						Guildford
Guildford																												GU2 5XH
GU2 5XH																												Tel: 01483 879222
Tel:	01483 876447 	Fax:	01483 300374
THE PRECiSE TRIAL
PREvention of Cancer by Intervention with Selenium
THE UK PILOT STUDY

CONTENTS	page
Introduction	1
Background	1
	Se and Cancer Incidence 	1
	Se and Cancer Prevention	2
	Anticipated Treatment Effect in PRECISE Trial Based on UK and 	2
	European Se Status		Effect of Se on Mood	3
Aim																																																	  3
Study Design	3
Study Population	4
Eligibility Criteria	4
Study Procedures	4
	Patient recruitment	4
	Allocation of treatment	4
	Contacting patients for the study	5
	Screening visit	5
	Run-in visit	5
	Entry to randomised controlled trial	5
	Follow up after the first six months of treatment	6
	Follow up every six months thereafter	6
	The end of the pilot study	6
	Timing of questionnaires	6
     Dose modifications																																							  7
	Assessment of compliance	7
Endpoints	7
	Principal end points	7
	Subsidiary end points	7
	Long-term end-points of the PRECISE Study	8
    Assessment of end-points																																			  8
Reporting Adverse Events	8
Statistical Considerations	8
References	9
Appendix 1 – Questionnaires	attached separately
	Screening visit
	Entry to Run-in
	Entry to Randomised Controlled Trial
	Six month follow up
	Twelve month follow up
	Eighteen month follow up
	Food-Frequency	
	POMS BI 
	SF-36 
Appendix 2 - Patient information and letters	attached separately
	Initial letter of invitation 
	Reminder letter
	Patient information sheet

Appendix 3 - Consent form	attached separately
	Consent for notesearch 
	Consent for entry into the trial

Appendix 4 - South Western Oncology Group	attached separately
		(SWOG) Grading Scale	
Introduction

There is a considerable body of evidence showing a protective effect of selenium (Se) against cancer which has recently been reinforced by the results of a number of important studies in the US and Finland. (1),(2),(3),(4) Thus there is concern that current levels of Se in the diet may be sub-optimal in terms of cancer protection.  Within the UK, dietary Se levels are considerably lower than in the US, and have fallen very significantly in recent years.  Levels now stand at around half the Government's defined Reference Nutrient Intake. (5) 

Confirmation of the viability of preventing cancer by using a dietary supplement of Se would have tremendous potential for reducing cancer incidence and mortality for many of the commonest forms of the disease at relatively low cost.

Supplementation with Se may confer an additional benefit.  A positive effect on mood has been reported in a number of studies.(6),(7),(8)


Background

No known cancer strategy to date has produced results comparable with those of the Nutritional Prevention of Cancer (NPC) trial which used organic selenium as the preventative agent.(1),(2)
This was a randomised, double-blind, placebo-controlled clinical intervention study, limited to patients (n = 1312) with a previous history of non-melanoma skin cancer.   Those patients who were randomised to 200 g/d of Se had significantly lower total cancer mortality (50%), total cancer incidence (37%), and a significant reduction in the incidence of lung (46%), colorectal (58%), and prostate cancer (63%) when compared to those in the placebo group.  The PRECISE Trial has been planned as a repeat of the NPC trial on an extended basis in different population groups and on a larger scale.   


Se and Cancer Incidence 

The extensive experimental evidence, reviewed by Medina and Morrison,(9) and Combs and Clark,(10) indicates that Se supplementation reduces the incidence of cancer in animals. There are over 100 published studies in 20 different experimental animal models of spontaneous, viral, and chemically induced tumours.  In two thirds of these studies, a significant reduction in tumour incidence was reported and in one third of these studies, a reduction of over 50% in tumour incidence was observed.  This literature indicates that Se supplementation in animals can reduce the incidence of skin, colorectal, breast, stomach, hepatic, oesophageal, oral, tracheal, pancreatic, kidney, and lung cancers.

Geographic studies have shown a consistent trend for populations with low Se levels to have a higher cancer mortality rate.  Epidemiological cancer studies in individuals have produced more variable results, but the trend in most studies is for individuals with lower Se levels to have a higher incidence of cancer.  A number of cohort and nested case-control studies have shown a lower risk of internal malignancies for individuals with higher plasma Se levels.  A recent meta-analysis of cohort studies comparing the effect of serum Se, retinol, beta-carotene, and vitamin E suggests that Se has a remarkably consistent protective effect.(11)  Other recent epidemiological studies showing the protective effect of Se have involved cancers of the bladder, ovaries,(12) pancreas,(13) thyroid,(14) lung and stomach.(15) Additional epidemiological designs have shown protective effects of Se for cancers of the oesophagus,(16) lung,(17),(18) skin (melanoma),(19) head and neck,(20) brain,(21) stomach(22) and a suggested effect for prostate cancer.(23),(24)  

Two recent studies have reinforced these findings.  In a nested case-control study within a
cohort of 9,000 Finnish individuals, the adjusted relative risk of lung cancer between the highest and lowest tertiles of serum Se was found to be 0.41,(4)  while the Health Professionals' Cohort Study demonstrated a strong inverse dose-response association between toenail Se and prostate cancer.(3)  This study showed a 65% reduction in the risk of advanced prostate cancer among men with the highest, as compared to the lowest, Se status (by quintile of toenail Se).


Se and Cancer Prevention

NCI sponsored trials in China for the prevention of oesophageal cancer observed a reduction in total cancer mortality in the intervention arm containing selenium, -carotene, and vitamin E.(25)  Other trials in China using Selenium as a single agent reported a reduced incidence of liver cancer.(26)  Recently, in a double-blind randomised clinical trial in Italy, a 44% reduction in the incidence of recurrent neoplastic polyps was observed for subjects in the group randomised to 200 g/day of Se plus other vitamins and minerals versus a placebo.(27) 

The Nutritional Prevention of Cancer (NPC) trial(1),(2) was the first trial conducted in a Western population, to show that a nutritional supplement could reduce the incidence of cancer.   The NPC trial was a double-blind, placebo-controlled, randomised cancer prevention trial, in which 1,312 patients with a history of non-melanoma skin cancer received a daily dose of 200 g of Se or a placebo for up to 10 years.  There was a statistically significant reduction of 37% (RR=0.63, 95% CI, 0.47-0.85) in total cancer incidence with a 200 g supplement of selenium.(1),(2)  There were 77 cancers in the treatment group versus 119 cases in the placebo group during the first ten-years of follow-up between 1983 and 1993.  A significant reduction in the incidence of lung (46%), colorectal (58%), and prostate cancer (63%) was observed.  A statistically significant 50% reduction in total cancer mortality was also observed in the Se-supplemented group, with 29 deaths in the treatment group and 57 deaths among controls (RR=0.50, 95% CI, 0.31-0.80). 

Analysis of treatment effect by baseline plasma Se level shows that the strongest treatment effect was observed in subjects who were classified in the bottom tertile of plasma Se(28) (i.e.<106 g/L) before dosing, when the trial was initiated.


Total Cancer Incidence 1983-96 by Baseline Plasma Se Level (28)
	
Se Level 
g/L	Se cases 	Placebo cases	RR	CI	P-value	
<106	28	56	0.52	0.33, 0.82	0.005	
106-121	34	49	0.64	0.40, 0.97	0.4	
>121	45	41	1.00	0.65, 1.54	0.99	


Anticipated Treatment Effect in PRECISE Trial Based on UK and European Se Status

Plasma Se levels below 106 g/L are now the norm in much of northern Europe (with the exception of Finland where Se is added to fertilizers) since the use of high Se wheat from North America for bread making has largely been replaced by low-Se European and UK varieties.(5) 

Subjects in the NPC trial had a Se intake of around 90g/day.(1)  Current dietary Se intake in the UK is around 34g/day(29) (much lower than the Government's defined Reference Nutrient Intake of 75 g/day for men and 60 g/day for women).(5)  The estimated mean intake of Se of adults in Sweden and Denmark is similarly low.(30),(31) 

Correspondingly low serum/plasma selenium levels have been found in these countries, particularly in the UK where values of 60-79 g/L were measured recently.(32),(33),(34)  Thus UK subjects and the great majority of those from the Danish and Swedish cohorts would fall into the bottom tertile of the NPC trial.  These Northern European populations are therefore ideal for testing the effect of Se supplementation, as the strongest treatment effect for cancer in the NPC trial was observed in those participants who had the lowest baseline Se levels.  The data from the low-Se countries can be compared to those study cohorts from the US and Finland where Se intakes and therefore status are higher but still moderate.


Effect of Se on Mood

A positive effect of Se on mood has been reported in three separate studies.(6),(7),(8) 


Aim

The aim of the PRECISE Trial is to test whether daily supplementation with the essential trace element Se will significantly reduce total cancer incidence and site-specific cancer incidence in the general population. 
The aim of the UK Pilot Study is to test the viability of carrying out the Precise trial within the UK. 

Prior to activating the full PRECISE Trial within the UK, the UK Pilot Study aims to:

i)	determine whether compliance will be satisfactory
ii)	determine the number of practices, subjects and staff needed for successful completion of subject recruitment and randomisation
iii)	determine the number of staff needed for successful data collection and analysis
iv)	test the questionnaires and the database management system specifically created for the PRECISE trial 
v)	identify potential logistical issues related to the trial protocol that need to be resolved prior to starting the full study
An additional aim of the pilot is to see if selenium supplementation has a beneficial effect on mood.

A pilot study is currently underway in Denmark


Study design

The PRECISE trial is planned as a randomised, double-blind, placebo-controlled cancer prevention trial, with an estimated duration of 5 years and with 52,000 subjects recruited from the general populations of five countries: the United Kingdom, Denmark, Sweden, Finland and the United States.  The UK Pilot Study will recruit a total of 510 subjects from three general practices in three areas of differing characteristics that are widely separated geographically. The pilot study has an estimated duration of two years.


Study Population

Subjects will be equal numbers of men and women, aged 60 to 74 years, registered with practices in the MRC General Practice Research Framework (GPRF). There will be equal numbers of subjects from each of the five year strata; i.e. 60 - 64; 65 - 69; 70 - 74. The age range selected for the trial is based on age-specific total cancer incidence rates.  Below age 60, the incidence is too low to yield enough cases for the trial.  Exclusion of subjects over 74 years of age is to avoid limitation of follow-up time after the initial five years of treatment by a high death rate from causes other than cancer. Additionally, the age range 60-74 showed the strongest treatment effect in the NPC trial.


Eligibility criteria

All those aged between 60 and 74 years are eligible except those:
1)	with a SWOG (Southwest Oncology Group grading scale) performance status score greater than 1 or equivalent (see appendix 4);
2)	with active liver or kidney disease (known abnormal liver or kidney function)
3)	with a prior diagnosis of cancer (excluding non-melanoma skin cancer);
4)	with diagnosed HIV infection;
5)	with diminished mental capacity (subjects must be able to give informed consent to participate as defined by ethics committees);
6)	taking 50 g/day or more of selenium supplements. 


Study procedures


Patient recuitment

The Danish pilot study has been in operation since November 1998. Based on their experience and following advice from Dr Madge Vickers of the MRC GPRF, it is hoped that 25% of those eligible by age will agree to be randomised into the study. Therefore it is estimated that 2040 subjects will be invited to take part in the study and 510 will be randomised.


Allocation of treatment

The pilot and PRECISE study will have four treatment groups: placebo, and 100, 200 or 300 g/day of Se supplementation. The intervention agent is the same high Se yeast (SelenoPreciseTM) used in the previous US trial.  The placebo is also yeast based, being composed of the same ingredients as the selenium tablets, but without selenium.  Blinding will be carried out at three stages making it impossible for an individual at any of the stages to reveal the code without alert.  The dose of 200 g/day of Se is necessary for replication of the results from the NPC trial.  The higher and lower doses will enable a dose-response relationship to be established.  All of these dosages are within the range of Se intakes observed in healthy free-living subjects in the US.(35),(36)


Contacting patients for the study

Following a note search by the MRC GPRF trial nurse, letters of invitation will be sent to eligible subjects in the GP practice.  Subjects will indicate their interest in the study by returning a reply slip for a screening visit.  If subjects do not respond to the initial invitation, a second letter will be sent one month later.  If there is still no response, it will be assumed that the subject does not wish to consider taking part in the study.


Screening visit

The nurse will make an appointment for the screening visit by telephone.  During this appointment the nurse will explain about the study, give the subject an information sheet and answer any questions the subject has.  She will ask the subject to sign a consent form for his or her medical records to be reviewed for the study.  She will also record the subject's name and date of birth in the screening log book which will generate a pre-dosing study number (screening number).  This log book will also be used to record reasons that subjects are not willing to enter either the run-in period or the entry to the randomised controlled trial.  


Run-in visit

Having been given two weeks to reflect following the screening visit, the subject will then enrol for a four-week placebo run-in period. The subject will sign a consent form stating that he/she is willing to enter the study.  Guided by the nurse, the subject will complete the "Entry to run-in" questionnaire and will be given the food-frequency questionnaire and instructions on how to fill it in at home. He/she will be also be given an envelope for toenail clippings (which can be used for Se analysis) and instructions on how to take the clippings just before the next scheduled visit.  He/she will be given a four-week supply of placebo tablets to take daily.


Entry to randomised controlled trial

At the end of the run-in period, a subject who has been deemed to be compliant by tablet count will be entered into the randomised control trial if he/she is willing.  He/she will complete the "Entry to Randomised Controlled Trial" questionnaire (baseline questionnaire) with the help of the nurse, the SF-36 questionnaire" and the "POMS-BI" questionnaire (this assesses mood which may be positively influenced by Se) and he/she will be randomised into the trial.  Random permuted blocks will be used to generate a randomised list at the Clinical Trials and Statistics Unit at the Institute of Cancer Research.  The MRC GPRF stockroom will supply the trial tablets to the practices according to this list.  The trial nurse will telephone the randomisation office at the Institute of Cancer Research and the subject is assigned a trial number.  The nurse will write the subject's name on the pack of tablets which bears the subject's trial number.

The nurse will take a baseline blood sample and separate it into different blood components.  The total amount of whole blood drawn will be 30-35 ml. Twenty-five ml will be drawn into anticoagulant tubes in order to separate the plasma and buffy coat.  Ten ml will be drawn into serum separator tubes to obtain serum.  From these blood draws, approximately 4 ml of serum, 6 ml of plasma, 2 ml of buffy coat, and 2 ml of red blood cells will be obtained giving a total of 14 aliquot tubes per subject.  Samples will be stored at the practice in a -200C freezer and will be posted fortnightly in dry ice to the -80o C storage facility at the University of Surrey.  (Half of the samples will be sent from Surrey on to a central storage facility in Denmark to avoid the possibility of losing all samples owing to a freezer failure.)  One plasma sample will be analysed for Se, while the others will be stored for later analyses such as that of vitamin E, which is known to compensate for Se deficiency in some situations.  The nurse will also collect the envelope containing the subject's toenail clippings and ensure that the food-frequency questionnaire has been properly completed.

Recruitment is expected to be achieved over a nine-month period and should be complete twelve months into the pilot, with the last six-month follow-up visit being achieved at 20 months.


Follow-up after the first six months of treatment
The subject will see the nurse for the first follow up appointment six months after  randomisation.  The nurse will telephone the subject two weeks before the appointment to confirm the appointment.  A blood sample (20ml) will be drawn and separated to give a plasma sample (four aliquots of 1ml) for Se analysis, and a "Follow-up" questionnaire, the "SF-36 Questionnaire" and the "POMS-BI" (mood assessment) questionnaire will be completed with the nurse.  The nurse will check the packaging from the previous six-month supply of tablets to check for compliance.  If satisfactory, she will give the subject a further six months supply of tablets.  

Follow up every six months thereafter
The subject will come back to see the trial nurse at six-monthly intervals until the pilot study ends.  He/she will complete a follow up questionnaire with the nurse at the clinic during each the six-monthly visit. The nurse will check the packaging from the previous six-month supply of tablets to check for compliance.  A further six-month supply of tablets will be given.  

The end of the pilot study
If the main study follows on from the pilot study, subjects will sign another consent form stating that they are happy to enter the main study.  The subject will continue to visit the trial nurse at six monthly intervals when the "Follow-up" questionnaire will be completed with the nurse and a further six-month supply of tablets given.  Subjects will receive letters from the practices informing them of whether or not the pilot will progress to a full clinical trial.

Timing of questionnaires
At initial screening 	Screening visit (eligibility) questionnaire	
At entry to Run-in 	Entry to run-in questionnaire
Food-frequency questionnaire (to take home)	
At entry to Pilot Study 	Entry to Randomised Controlled Trial (baseline questionnaire)
SF-36 questionnaire
POMS-BI (mood assessment)	
At 6-month follow up
	Six month Follow-up questionnaire 
SF-36 questionnaire
POMS-BI (mood assessment)	
6 monthly thereafter	Follow-up questionnaires	

All questionnaires except the Food-frequency questionnaire will be completed in the clinic by the subject.


Dose modifications

In the event of any subjects becoming ill with new, serious illness, treatment will be discontinued until the medical condition has stabilised, and will be restarted once the subject has recovered or has been discharged from hospital.  In the event of subjects requiring kidney dialysis, treatment will be discontinued because of the importance of the kidneys in the excretion of selenium.  If subjects are concerned about the possibility of a minor illness being related to their treatment supplement, they should stop taking the supplement for two weeks and consult with their GP or trial nurse before restarting.  Any persistent problems should be referred to the GP for evaluation. 


Assessment of compliance

As compliance is the primary outcome measure in the UK pilot study, any problems with compliance will have been identified and measures taken to deal with them before initiation of the full trial. The four-week placebo run-in period should help to eliminate those who are non-compliant.
Compliance will be assessed by pill-count (tablets are packed in blister packs labelled with the days of the week) and by analysis of Se in plasma.  Subjects will have blood sampled for Se analysis at the entry to the run-in period and at the first six-month follow up visit.  Toenail clippings, which may also be analysed for Se, will be collected at randomisation.

Comparison of plasma Se levels at the first six-monthly follow-up visit with initial levels will enable a check to be made on “drop-ins” (possible because of the availability of Se supplements over-the-counter) as well as "drop-outs".  It is estimated that the last follow-up visit will take place at 20 months. 

To encourage compliance, subjects will receive six-monthly newsletters.  As there is no history of side effects of selenium at the dosages used, and a three in four chance of getting active supplement, compliance is expected to be good.  On the basis of previous experience in the NPC trial, the drop-out rate is envisaged to be no more than 4%, though it may be lower owing to recruitment through general practices and the relationship of subjects with their primary-care physician.


Endpoints

Principal end points
Extent of compliance
Rates of recruitment and randomisation
Proportion of withdrawals from study at six-month follow-up

Subsidiary end points
Time required for each trial-related activity within the practices
Satisfactory performance of questionnaires
Satisfactory data entry and management system
Improvement in mood


Long-term end-points of the PRECISE Study
The subjects of the pilot will become part of the main PRECISE trial when activated.  Thus the primary end-point of cancer incidence and the secondary end-points of cancer mortality, incidence of other major illnesses and hospitalisations will also be documented within the pilot, although no significant findings can be ascertained at this point. 


Assessment of end-points
·	Compliance will be estimated by:
(i) comparison of plasma Se at the six-monthly follow-up visit with initial levels (to identify “drop-ins” as well as “drop-outs”) 
(ii) remaining pill count (pills are blister-packed in four rows of seven, marked with days of the week)
·	Rates of recruitment and randomisation and proportion of withdrawals from study at the follow-up visits will be calculated from the numbers of subjects entering and remaining at the appropriate stage of the trial.  
·	Time required for each trial-related activity within the practices will be calculated from numbers of subjects dealt with over a measured period for each activity.  
·	Compliance with, and comprehension of, questionnaires, will be monitored through trial nurses, experienced in their administration.  
·	Satisfactory data entry and management system will be judged by the trial statistician.  
·	Cancer incidence and the incidence of other major illnesses will be determined by a number of methods:- flagging of patients' notes at surgeries; questioning by nurse at follow-up visit; annual note search; inspection of hospital discharge letters; obtaining pathology reports.  
·	Cancer mortality will be determined by a number of methods:- flagging of patients' notes; obtaining pathology reports; flagging patients at the NHS Central Registry and obtaining a copy of the death certificate.
·	Mood  will be investigated by means of a mood-state questionnaire at the Run-in visit and again at the first six-monthly follow up visit.


Reporting Adverse Events

Subjects will be monitored for signs of toxicity at six-monthly follow up visits.  The doses used in the study are well within the safe and normal dietary range in many countries and are therefore unlikely to produce and toxic effects.  Any episodes of possible selenium toxicity or other trial-related injury should be reported immediately to the subject's GP. Serious adverse events should be reported immediately to the GP who will contact Professor Hilary Thomas (on 01483 406823).  She in turn will make them known to Professor Larry Clark, the Principal Investigator of the PRECISE Trial.


Statistical Considerations

The proposed sample size for the pilot is 510 subjects, recruited through three practices.  The sample size has been chosen to give a large enough number of subjects (170) in each practice from which to be able to draw reasonable inferences about recruitment, compliance and loss-to-follow-up, while keeping the cost within reasonable bounds.  The use of practices of differing characteristics from different areas of the country will improve the degree of confidence in the outcome measures.  The main trial will have a total of 52,000 subjects, with 8,300 subjects from each of the following four countries; the UK, Sweden, Finland and Denmark and 18,800 subjects from the USA.  This will have at least 90% statistical power to detect a 14% reduction in total cancer incidence during the initial five-year period of treatment and follow up.

References

1. Clark LC, Combs GF Jr, Turnbull BW, Slate EH, Chalker DK, Chow J, Davis LS, Glover RA, Graham GF, Gross EG, et al. Effects of selenium supplementation for cancer prevention in patients with carcinoma of the skin. A randomized controlled trial. Nutritional Prevention of Cancer Study Group [see Comments] [published erratum appears in JAMA 1997 May 21;277(19):1520]. JAMA 1996 Dec 25;276(24):1957-63.
Comment in: JAMA 1996 Dec 25;276(24):1984-5
	Comment in: JAMA 1997 Mar 19;277(11):880; discussion 881

2. Clark LC, Dalkin B, Krongrad A, et al., Decreased Incidence of Prostate Cancer with Selenium Supplementation: Results of a Double-Blind Cancer Prevention Trial. Br J Urology 1998, 81:730-34.

3. Yoshizawa K, Willett WC, Morris SJ, Stampfer MJ, Spiegelman D, Rimm EB, Giovannucci E, Toenail selenium as a predictor for prostate cancer incidence.  J Natl Cancer Inst 1998, 90:1219-1224.

4. Knekt P, Marniemi J, Teppo L, Heliovaara M, Aromaa A, Is low selenium status a risk factor for lung cancer? Am J Epidemiol 1998, 148, 975-82.

5. Rayman M P, Dietary selenium: time to act. BMJ, 1997; 314:387-388

6. Benton D & Cook R, The impact of selenium supplementation on mood, Biol. Psy. 29, 1092-8 (1991)

7. Hawkes W C & Hornbostel L, Effects of dietary selenium on mood in healthy men living in a metabolic research    
    unit, Biol. Psychiatry, 39, 121-8 (1996)

8. Finlay J W & Penland J G, Adequacy or deprivation of dietary selenium in healthy men: Clinical and 
    psychological findings, Tr. El. in Exptl. Med 11:11-27 (1998)

9. Medina, D, Morrison D G, Current Ideas on Selenium as a Chemopreventive Agent. Pathol Immunopathology 1988; 7:187-99.

10. Combs GF, Clark L. Selenium and Cancer Prevention. In:Antioxidant Nutrients and Disease Prevention.: CRC Press, Boca Raton, NY.; 1997 Apr.

11. Comstock, G, Bush, T, Helzlsouer K, Serum Retinol, Beta-Carotene, Vitamin E, and Selenium as Related to Subsequent Cancer of Specific Sites. Am J of Epidemiol 1992;135:115-20.

12. Helzlsouer, Kathy J.; Comstock, George W.; Morris, J. .Selenium, Lycopene, alpha Tocopherol, beta-Carotene, Retinol, and Subsequent Bladder Cancer. 1989 Nov; 49:6144-8.

13. Burney, P. G. J.; Comstock, G. W.; Morris, J. S. Serologic Precursors of Cancer: Serum Micronutrients and the Subsequent Risk of Pancreatic Cancer. Cancer 1989; 49: 895-900.

14. Glattre, E.; Thomassen, Y.; Thoresen, S. O., et al Prediagnostic Serum Selenium in a Case-Control Study of Thyroid Cancer.Int J of Epidemiol 1989; 18(1):45-9
	
15. Knekt, Paul; Aromaa, Arpo; Maatela, Jouni, et al .Serum Selenium and Subsequent Risk of Cancer Among Finnish Men and Women. JNCI 1990; 82:864-8.
	
16. Jaskiewicz, K.; Marasas, F. O.; Rossouw, J. E., et al .Selenium and Other Mineral Elements in Populations at Risk for Esophageal Cancer. Cancer 1988; 62:2635-9.

17. Gerhardsson, L.; Brune, D.; Nordberg, I. G. F., et al .Protective Effect of Selenium on Lung Cancer in Smelter Workers. Brit J of Industrial Med 1985; 42: 617-26.

18. Miyamoto, H.; Araya, Y.; Ito, M., et al. Serum Selenium and Vitamin E Concentrations in Families of Lung Cancer Patients. Cancer 1987 Sep; 60:1159-62.

19. Reinhold, U.; Blitz, H; Bayer, W, Schmidt KH, . Serum selenium levels in patients with malignant melanoma. Acta Dermato-Venereol, 69:132-6

20. Westin, Thomas; Ahlbom, Eva; Johansson, Elisabeth, et al. Circulating Levels of Selenium and Zinc in Relation to Nutritional Status in Patients With Head and Neck Cancer. Arch Otolaryngol Head Neck and Surg 1989 Sep; 115:1079-82.

21. Philipov P TK. Selenium concentrations in serum of patients with cerebral and extracerebral tumors. Zenralblatt Fur Neurchirurgie 1988;49(4):344-7.

22. Caygill CP, Lavery K, Judd PA, Hill MJ, Diplock AT. Serum selenium and gastric cancer in two regions of Norfolk. Food Additives & Contaminants 1989;6(3):359-63.
	
23. Criqui MH, et al. Selenium, retinol, retinol-binding protein, and uric acid.  Associations with cancer mortality in a population-based prospective case-control study. Ann Epidemiol 1991;1(5):385-93.

24. Ip, Clement; White, G.  Mammary cancer chemoprevention by inorganic and organic selenium: single agent treatment or in combination with vitamin E and their effects on in vitro immune functions. Carcinogenesis. 1987, 8:1763-66.

25. Blot, W.; Li, J.; Taylor, P., et al. Linxian Nutrition Intervention Trials. III.  Mortality and cancer incidence following supplementation with specific vitamin/mineral combinations in the Linxian general population.  JNCI 1-32.

26. Yu, Shu-Yu; Zhu, Ya-Jun L. W-G.; Huang, Qi-Sheng, et al .A Preliminary Report on the Intervention Trials of Primary Liver Cancer in High-Risk Populations with Nutritional Supplementation of Selenium in China. J of Biol Trace Elem Research 1991; 29:288-95.

27. Bonelli, L.; Conio, M.; Massa, P, et al. Chemoprevention with Antioxidants of Metachronus Adenomas of the Large Bowel. Cancer Prevention & Control 1998, 100: A351.

28. Clark LC, Turnbull BW, Slate EH et al., Subgroup analysis of the Nutritional Prevention of Cancer with Selenium trial.  Manuscript prepared for publication.

29. MAFF Food Surveillance Information Sheet, Oct. 1997, No 126

30. Johnsson L, Akesson B. A. Availability of selenium from soils in relation to human nutritional requirements in Sweden. Stockholm, Sweden: Swedish Environmental Protection Agency; 1997

31. Suadicani P, Hein HO, Gyntelberg F. Strong mediators of social inequalities in risk of ischaemic heart disease: a six-year follow-up in the Copenhagen Male Study. Int J Epidemiol 1997 Jun;26(3):516-22.

32. Barrington JW, Taylor M, Smith S and Bowen-Simpkins PJ, Selenium and recurrent miscarriage. J Obs Gyn 1997, 17:199-200

33. Shortt CT, Duthie GG, Robertson JD, Morrice PC, Nicol F, Arthur JR, Selenium status of a group of Scottish Adults. Eur J Clin Nutr 1997 51:400-4

34. Scott R, MacPherson A, Yates RWS et al. The effect of oral selenium supplementation on human sperm motility. Br. J. Urology 1998, 82:76-80

35. Whanger P, Vendeland S, Park Y-C, Xia Y. Metabolism of sub-toxic levels of selenium in animals and humans.   
	Ann Clin Lab Sci 1996, 26:99-113

36. Longnecker MP, Taylor PR, Levander OA, et al. Selenium in Diet, Blood and Toenails in Relation to Human Health in a Seleniferous Area. Am J of Clin Nutr 1991; 53(5):1288-94


APPENDICES (attached separately)

1.	Questionnaires
Screening visit
Entry to Run-in
Entry to Randomised Controlled Trial
Six month follow up
Twelve month follow up
Eighteen month follow up
Food Frequency 
POMS-BI
SF-36 Quality of Life

2.	Patient information and letters
Initial letter of invitation 
Reminder letter
Patient information sheet

3.	Patient consent forms
Consent for note search
Consent to enter study

4.	South Western Oncology Group (SWOG) Grading Scale 
