# Supplementary material for: A Randomized Trial of Selenium Supplementation and Risk of Type-2 Diabetes, as Assessed by Plasma Adiponectin
Source: PLoS One. 2012 Sep 19;7(9):e45269. doi: 10.1371/journal.pone.0045269 (PMC3446875; doi:10.1371/journal.pone.0045269)
Supplement: Table S1 — Cross-sectional association between plasma selenium and adiponectin concentrations at baseline by sex. (DOC) [file pone.0045269.s003.doc]

**Table S1.** Cross-sectional association between plasma selenium and adiponectin concentrations at baseline by sex*

|  |  | **Quartile of baseline selenium level (ng/g)** | | | |  |
| --- | --- | --- | --- | --- | --- | --- |
|  | **50-ng/g increase in baseline selenium level** | **First (48.6 to 75.0)** | **Second (75.1 to 88.0)** | **Third (88.1 to 100.0)** | **Fourth (100.1 to 177.0)** | ***P* value for trend†** |
| **Men** |  |  |  |  |  |  |
| Participants (n) | 216 | 53 | 54 | 67 | 42 |  |
| **Baseline adiponectin level (µg/mL)** |  |  |  |  |  |  |
| Geometric mean (SD) | 6.32 (1.90) | 6.51 (1.67) | 6.43 (1.94) | 6.61 (1.75) | 5.55 (2.33) |  |
| Geometric mean ratio (95% CI) | 0.91 | 1 | 0.96 | 0.92 | 0.81 | 0.07 |
|  | (0.74 to 1.13) | (Reference) | (0.78 to 1.18) | (0.76 to 1.13) | (0.65 to 1.02) |  |
| **Women** |  |  |  |  |  |  |
| Participants (n) | 203 | 53 | 52 | 38 | 60 |  |
| **Baseline adiponectin level (µg/mL)** |  |  |  |  |  |  |
| Geometric mean (SD) | 10.24 (1.62) | 10.58 (1.60) | 10.40 (1.47) | 10.12 (1.59) | 9.88 (1.80) |  |
| Geometric mean ratio (95% CI) | 0.89 | 1 | 1.04 | 0.97 | 0.90 | 0.23 |
|  | (0.73 to 1.07) | (Reference) | (0.84 to 1.28) | (0.77 to 1.22) | (0.73 to 1.10) |  |

* Results were obtained from linear regression models on log-transformed baseline adiponectin levels with interactions between baseline selenium levels and sex, and adjusted for age (continuous), study center (Bungay, Guisborough, Bromsgrove, or Linthorpe), smoking status (never, former, or current), drinking habits (never, former, or current), body mass index (continuous), waist circumference (continuous), baseline total cholesterol level (continuous), baseline HDL cholesterol level (continuous), use of lipid lowering medications, and use of diabetes medications.

† *P* values for linear trend were obtained from Wald tests for the coefficient of an ordinal variable with the median baseline selenium level of each quartile in linear regression models.
