# Supplementary material for: A Randomized Trial of Selenium Supplementation and Risk of Type-2 Diabetes, as Assessed by Plasma Adiponectin
Source: PLoS One. 2012 Sep 19;7(9):e45269. doi: 10.1371/journal.pone.0045269 (PMC3446875; doi:10.1371/journal.pone.0045269)
Supplement: Table S2 — Effect of selenium supplementation on changes in plasma adiponectin concentrations after six months by sex. (DOC) [file pone.0045269.s004.doc]

**Table S2.** Effect of selenium supplementation on changes in plasma adiponectin concentrations after 6 months by sex*

|  |  | **Selenium dose (µg/d)** | | |  |
| --- | --- | --- | --- | --- | --- |
|  | **Placebo** | **100** | **200** | **300** | ***P* value†** |
| **Men** |  |  |  |  |  |
| **Plasma adiponectin level (µg/mL)** |  |  |  |  |  |
| Geometric mean (SD) at baseline | 7.07 (1.82) | 5.53 (2.12) | 5.95 (1.77) | 7.15 (1.85) |  |
| Geometric mean (SD) at 6 mo | 7.03 (1.92) | 4.99 (2.41) | 6.04 (1.83) | 7.29 (1.75) |  |
| Relative ratio (95% CI) | 1 | 0.89 | 1.04 | 1.03 | 0.47 |
|  | (Reference) | (0.70 to 1.12) | (0.83 to 1.30) | (0.81 to 1.31) |  |
| **Women** |  |  |  |  |  |
| **Plasma adiponectin level (µg/mL)** |  |  |  |  |  |
| Geometric mean (SD) at baseline | 9.55 (1.74) | 10.21 (1.48) | 10.44 (1.55) | 11.32 (1.74) |  |
| Geometric mean (SD) at 6 mo | 9.60 (1.94) | 10.89 (1.54) | 10.09 (1.60) | 11.38 (1.65) |  |
| Relative ratio (95% CI) | 1 | 1.04 | 0.95 | 0.98 | 0.85 |
|  | (Reference) | (0.83 to 1.31) | (0.75 to 1.19) | (0.77 to 1.24) |  |

* Results were obtained from linear mixed models on log-transformed adiponectin levels with fixed interaction terms among time, treatment group, and sex, and random between-subject variations in both baseline adiponectin levels (intercepts) and adiponectin changes over time (slopes).

† Overall *P* value comparing the three active treatment groups to placebo within each sex, as obtained from the joint Wald test for all sex-specific treatment-by-time interaction coefficients in linear mixed models.
